# An experimental animal model for percutaneous procedures used in trigeminal neuralgia

**DOI:** 10.1007/s00701-017-3162-8

**Published:** 2017-04-10

**Authors:** Johannes Herta, Wei-Te Wang, Romana Höftberger, Sabine Breit, Sibylle Kneissl, Helga Bergmeister, Heber Ferraz-Leite

**Affiliations:** 1 Department of Neurosurgery, Medical University of Vienna, Vienna, Austria; 2 Institute of Neurology, Medical University of Vienna, Vienna, Austria; 3 Department of Pathobiology, University of Veterinary Medicine, Vienna, Austria; 4 Diagnostic Imaging, Department for Companion Animals and Horses, University of Veterinary Medicine, Vienna, Austria; 5 Department of Biomedical Research, Medical University of Vienna, Vienna, Austria

**Keywords:** Animal model, Trigeminal neuralgia, Balloon compression, Glycerol rhizolysis, Thermocoagulation, Percutaneous procedures

## Abstract

**Object:**

This study describes an experimental rabbit model that allows the reproduction of percutaneous operations that are used in patients with trigeminal neuralgia (TN). Attention was given to an exact anatomical description of the rabbit’s middle cranial fossa as well as the establishment of conditions for a successful procedure.

**Methods:**

Morphometric measurements were taken from 20 rabbit skulls and CT scans. The anatomy of the trigeminal nerve, as well as its surrounding structures, was assessed by bilateral dissection of 13 New Zealand white rabbits (NWR). An ideal approach of placing a needle through the foramen ovale to reach the TG was sought. Validation of correct placement was realized by fluoroscopy and confirmed by dissection.

**Results:**

Precise instructions for successful reproduction of percutaneous procedures in NWR were described. According to morphological measurements, for balloon compression of the trigeminal ganglion (TG) the maximal diameter of an introducing cannula is 1.85 mm. The diameter of an empty balloon catheter should not exceed 1.19 mm, and the length of the inflatable part of the balloon can range up to 4 mm. For thermocoagulation the needle electrodes must not exceed an external diameter of 1.39, mm and the length of the non-insolated tip can range up to 4 mm. Glycerol rhizolysis can be achieved because the trigeminal cistern in the NWR is a closed space that allows a long dwelling time (>10 min) of the contrast agent.

**Conclusions:**

An experimental NWR model intended for the reproduction of percutaneous procedures on the TG has been meticulously described. This provides a tool that enables further standardized animal research in the field of surgical treatment of TN.

**Electronic supplementary material:**

The online version of this article (doi:10.1007/s00701-017-3162-8) contains supplementary material, which is available to authorized users.

## Introduction

In trigeminal neuralgia (TN), surgical therapies are applied if medication is ineffective or if adverse effects exceed the benefits of drugs [5]. Besides microvascular decompression introduced by Jannetta in 1967, less invasive procedures with the aim of adding a lesion to the trigeminal ganglion (ganglion trigeminale, TG) are commonly used: percutaneous balloon compression (BC), percutaneous radiofrequency-thermocoagulation (TC) and percutaneous glycerol rhizolysis (GR) [6, 9, 14, 19]. All three procedures share the same approach to the TG. A cannula is inserted percutaneously near the corner of the mouth, and by means of X-ray fluoroscopy, controlled advancement to the foramen ovale (FO) is thereby made possible. Subsequently, the TG and/or the trigeminal nerve root is partly damaged by compression, thermocoagulation or toxicity of glycerol.

Despite the frequent use of these surgical procedures, little is known about the arising pathophysiological changes in the trigeminal system. To reveal these changes, several attempts have been made to create animal models. But experimental setups that came close to operations in humans were rare, and the majority of experiments were conducted on and around peripheral nerves and not at the TG [4, 7, 13, 15, 17, 18, 20]. Therefore, dogs were used only once to reproduce GR and TC, while New Zealand white rabbits (NWR) were used in three experimental studies for BC. Noteworthily, histological findings have been partly contradictory and a standardization of the experiments is still needed [3, 8, 11, 12, 16].

Preul et al. [16] used a rabbit model to simulate percutaneous procedures on the TG. But a thorough description of the anatomy as well as the used materials and methods is necessary to use their model. The present study aims to create a manual for reproducible percutaneous operations on the TG in NWR in order to unify further research. The conditions for a successful procedure were derived after dissections and morphometric measurements.

## Methods

After a preliminary morphometric study, we concluded that rabbits seem to be the most suitable animal to reproduce percutaneous procedures at the TG. Not only is the anatomical accessibility to the TG feasible, but also costs are moderate and the Animal Welfare Act allows experimentation with rabbits in most European countries.

### Preliminary CT measurements

Twenty cranial CT scans, performed in rabbits with toothache at the department of Diagnostic Imaging, University of Veterinary Medicine, Vienna, were reviewed to measure the size of their 40 foramina ovalia and other related anatomical landmarks (Table 1). Images were acquired by a Somatom Emotion 16 Scanner (Siemens). The multi-slice data set was made with a thickness of 750 μm and evaluated on a Multimodality Workplace (Syngo, Siemens). To compensate for different skull sizes, a linear regression analysis was conducted to find a relationship between the length of the skull defined by the distance between the external occipital protuberance (protuberantia occipitalis externa, EOP) and the incisive bone (os incisivum), as well as the weight of the animals.

**Table 1:** CT measurements taken from 20 rabbits (2.10 ± 0.78 kg)

Measure	Landmark	Plane	Orientation/description	Results
Distance	Left to right facial tuberosity	X1	Inner surface; most rostral point	31.85 ± 3.17 mm
Distance	Left to right jugular process	X2	Inner surface; most rostral point	24.33 ± 2.86 mm
Distance	Left to right FO	X2	Median of both foramina	8.35 ± 0.79 mm
Distance	FO to OF	X1	FO rostral surface; OF caudal surface	6.19 ± 1.05 mm
Distance	FOR to TNC	X1	FOR caudal surface; TNC rostral surface	8.36 ± 1.01 mm
Distance	FO to mandibular angle	X2	Median surface FO; lower, median surface angulus mandibulae	33.75 ± 7.78 mm
Distance	FO to FAC	X1	FAC caudal surface, FO rostral surface,	5.27 ± 0.91 mm
Distance	EOP to incisive bone (PI-line)	X1	Most distant points in the midline	87.16 ± 11.07 mm
Diameter	FO	X2 tilted to be perpendicular to the foramen	Inscribed circle of the FO (triangle shape)	2.13 ± 0.28 mm
Diameter	TNC	X2 tilted to be perpendicular to the canal	Inscribed circle of the TNC (oval shape, width used)	2.32 ± 0.37 mm
Angle	Angle of puncturing needle	X3	Entry point = caudo-median surface of the mandibular angle, target = caudal surface of the FO	73.23 ± 12.23°

FO = foramen ovale, OF = orbital fissure, TNC = trigeminal nerve canal, FAC = foramen alare caudale, EOP = external occipital protuberance, X1 = dorsoventral, X2 = transverse, X3 = sagittal

### Anatomical measurements under the microscope and histological localization of the trigeminal ganglion

**Fig. 1:**
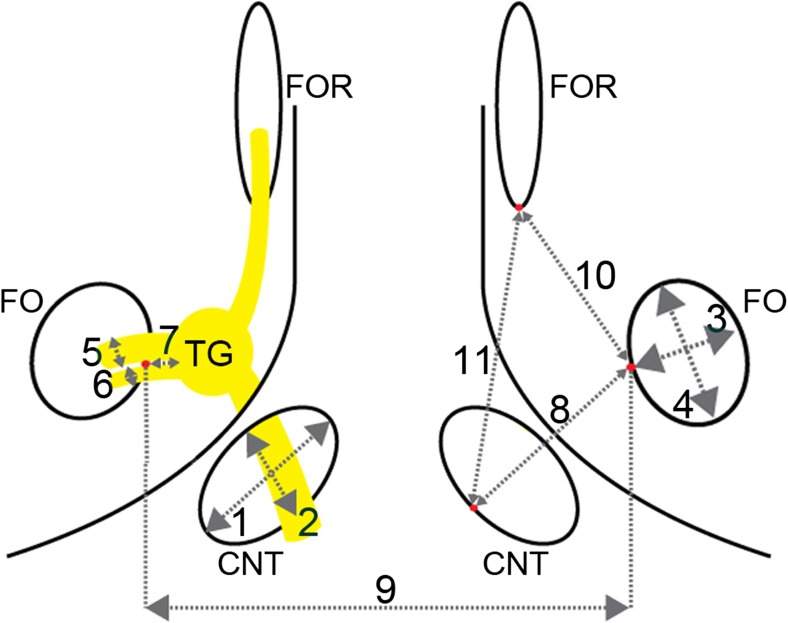
Schematic illustration of the middle cranial fossa in a dorsal view. Measurements, which were taken in the middle cranial fossa of 13 New Zealand white rabbits, are illustrated. Width of TNC (1) and FO (3), height of TNC (2) and FO (4), diameter of V3 (5) and V3 motor branch (6), distance between: medial limit FO and lateral limit TG (7), medial limit FO and medial limit TNC (8), medial limit FO and medial limit FO (9), caudal limit OF and medial limit FO (10) and caudal limit OF and medial limit TNC (11). FO, foramen ovale; TNC, trigeminal nerve canal; V3, mandibular nerve; TG, trigeminal ganglion; OF, orbital fissure

Thirteen heads of NWRs with a mean weight of 2.10 ± 0.78 kg were dissected to obtain measurements of anatomical structures and their relationships in the middle cranial fossa. All rabbits had been used and killed in previous animal experiments with a valid approval of the Commission for Animal Experimentation, leaving the skull and the brain intact. Dissection was performed in the operating rooms of the Department of Biomedical Research. Measurements, illustrated in Fig. 1, were performed under an operating microscope with a digital vernier caliper (Kinzo; Type: 12W68). Besides that, eight intracranial trigeminal nerves of four animals were harvested, inlaid in 10% formalin and further processed at the Institute of Neurology for histological confirmation of the localization of the TG. Each probe was cut in multiple sections [brainstem, trigeminal nerve root, TG, mandibular nerve (n. mandibularis, V3), maxillary nerve (n. maxillaris, V2) and ophthalmic nerve (*N. ophthalmicus*, V1)] and subsequently stained with HE, SMI31, SSB, CD68 and toluidine blue.

### Simulation of percutaneous procedures in cadavers

A percutaneous placement of a cannula through the cheek to reach the FO, as done in humans, is not possible in rabbits. For this reason, we switched to a submandibular approach and performed 30 percutaneous procedures (20 TC, 6 BC, 4 GR) in 16 NWR cadavers. Optimal needle placement was ensured by radiographs and subsequent cranial dissections. Because we aimed to provide a manual for experimental percutaneous procedures, the methods and used equipment are described in the results section.

## Results

### CT measurements

The mean weight of the rabbits was 2.10 ± 0.78 kg. CT measurements are listed in detail in Table 1. The mean inscribed circle radius of the FO was measured to be 2.13 ± 0.28 mm, while the mean inscribed circle radius of the trigeminal nerve canal (canalis nervi trigemini, TNC) was 2.32 ± 0.37 mm. There was a correlation between the radius of the FO and the weight of the rabbits (*r* = 0.54) as well as the mean distance between EOP and incisive bone (*r* = 0.67), left and right facial tuberosity (*r* = 0.65) and left and right jugular process (*r* = 0.44).

The trajectory of a puncturing cannula with the entry point at the anterior border of the mandibular angle and the FO as a target has an angle of 44.1 ± 3.21° in relation to the dorsoventral plane (×1). Accordingly, in relation to the transverse plane (×2), an angle of 73.23 ± 12.23° was measured. The cannula must be at least 41.53 mm long to reach the FO as shown by the distance between the FO and mandibular angle.

### Anatomical dissection, measurements and histological verification of the trigeminal ganglion

**Fig. 2:**
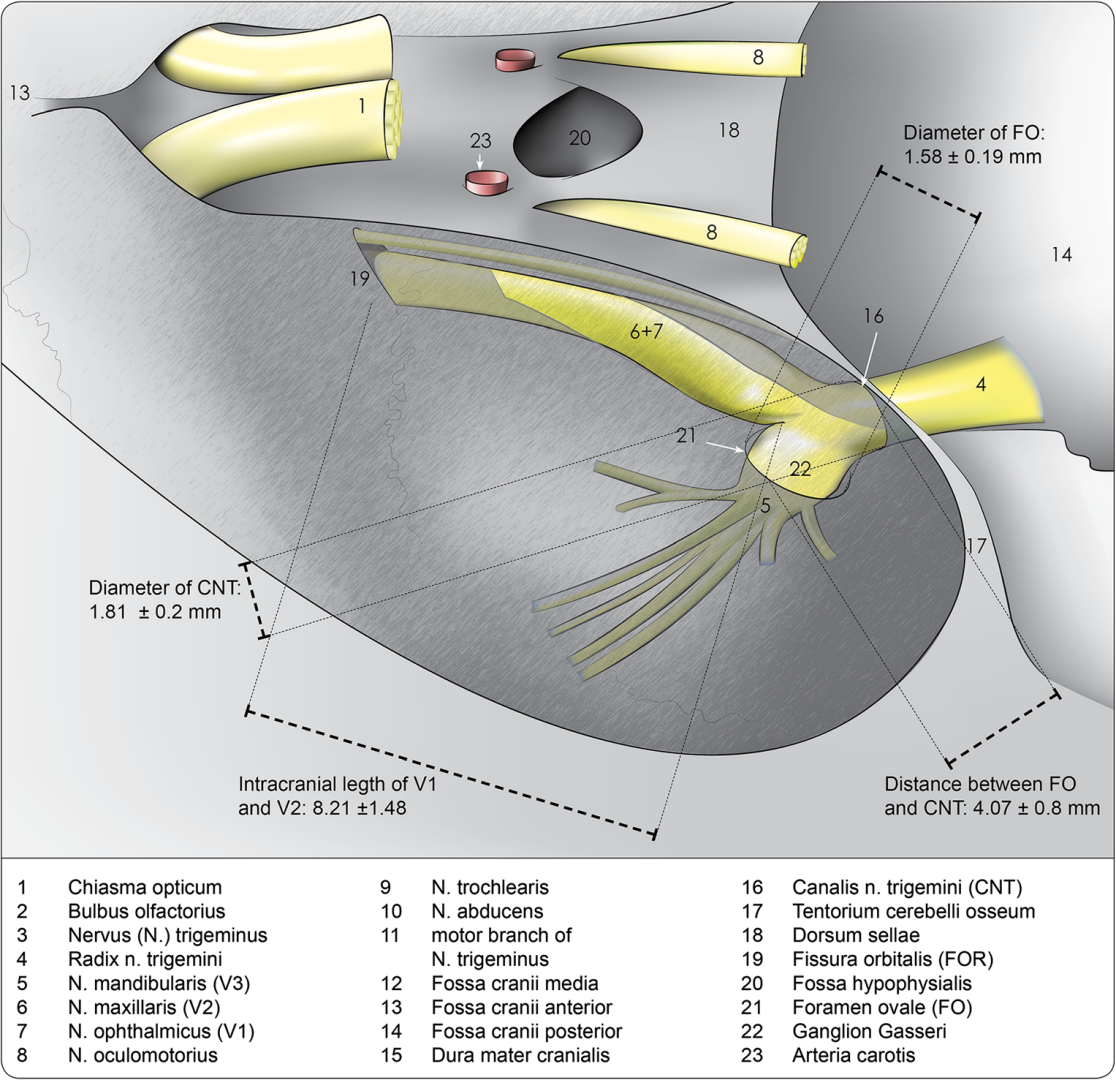
Anatomic schema and measures of middle cranial fossa structures, carried out by bilateral dissections in 13 New Zealand white rabbits

Anatomical dissections were performed in 13 NWRs with a mean weight of 4.09 ± 0.6 kg. Detailed illustrations of dissections are available online as Supplemental material. Accordingly, Fig. 2 not only serves as a legend but illustrates the anatomical relationships by giving morphometric measurements of the middle cranial fossa and the TG in NWR.

The FO had a mean length of 2.68 ± 0.33 mm and a mean width of 1.58 ± 0.19 mm. Thus, the diameter of a puncturing cannula must be smaller than 1.39 mm, equivalent to an 18-G needle. The TNC had a length of 1.81 ± 0.20 mm and a width of 3.23 ± 0.40 mm. The risk of puncturing the brainstem through the TNC is nearly zero because of an obtuse angle with the FO. The distance between the FO and the orbital fissure (OF) is representative for the intracranial length of V1 and V2, which leave the cranial cavity together through the OF. The distance between the FO and the TNC of approximately 4 mm indicates the needed length for compressing balloons or coagulating electrode tips.

Histological examination of eight TG and corresponding nerves in rabbits showed a similar localization to humans. No signs of damage due to manipulation could be seen.

### Conditions for a successful procedure

The following conditions should be fulfilled for a successful procedure in NWR.

*Percutaneous radiofrequency thermocoagulation (TC)*:

- Needle electrodes for TC should not exceed a diameter of 1.39 mm. A cannula smaller than 18 G should be used.
- The length of the non-insulated part of the needle electrode should not exceed a length of 3.27 mm.
- The length of the puncturing cannula needs to be longer than 45 mm.

*Percutaneous balloon compression (BC)*:

- An inserting cannula for BC should have a maximal diameter of 16 G. According to the measures obtained with dissections and CT scans, an outer diameter size between 1.39 mm and 1.85 mm is needed to reach slightly into the FO.
- The possible diameter for a deflated balloon is depending on the inserting cannula used. A normal 18-G needle has an inner diameter of approximately 0.838 mm; a 16-G needle has an inner diameter of 1.194 mm. However, there are also “thin wall” needles with a wider inner diameter available.
- The length of the inserting cannula needs to be longer than 45 mm.
- Balloon size should not exceed the distance between the FO and TNC. This means a balloon should be smaller than 4.07 ± 0.8 mm long.

*Percutaneous glycerol rhizolysis (GR)*:

- The puncturing needle should be longer than 45 mm and should not exceed an outer diameter of 18 G.
- The trigeminal cistern (TCI) is a closed cavity in NWR. The anatomical circumstances are very tight, and only small amounts of glycerol can be injected.

### Puncture technique and procedures

#### Positioning and orientation

**Fig. 3:**
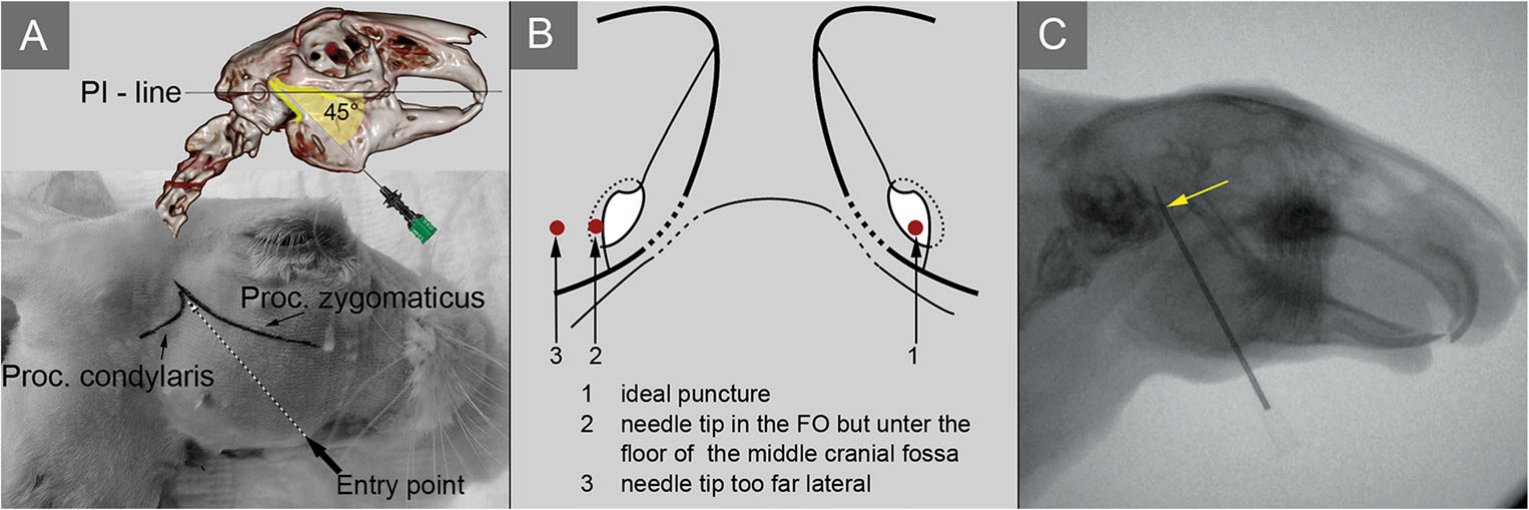
A: Important landmarks to reach the FO with a cannula. B: Common possibilities of false needle placement on the left. If the needle is correctly placed through the FO (B, right side) there is still a risk of perforating the dura mater and the temporal lobe (C, arrow)

The rabbit is placed in a prone position with a rotated head. The head is shaved, and landmarks are marked as shown in Fig. 3A. In the lateral view, the FO projects approximately at the intersection of the lower rim of the zygomatic arch and the rear rim of the condylar process (processus condylaris). The entry point for the cannula is laterally projected at the prominence of the mandibular angle.

#### Puncture procedure (Fig. 3A)

**Fig. 4:**
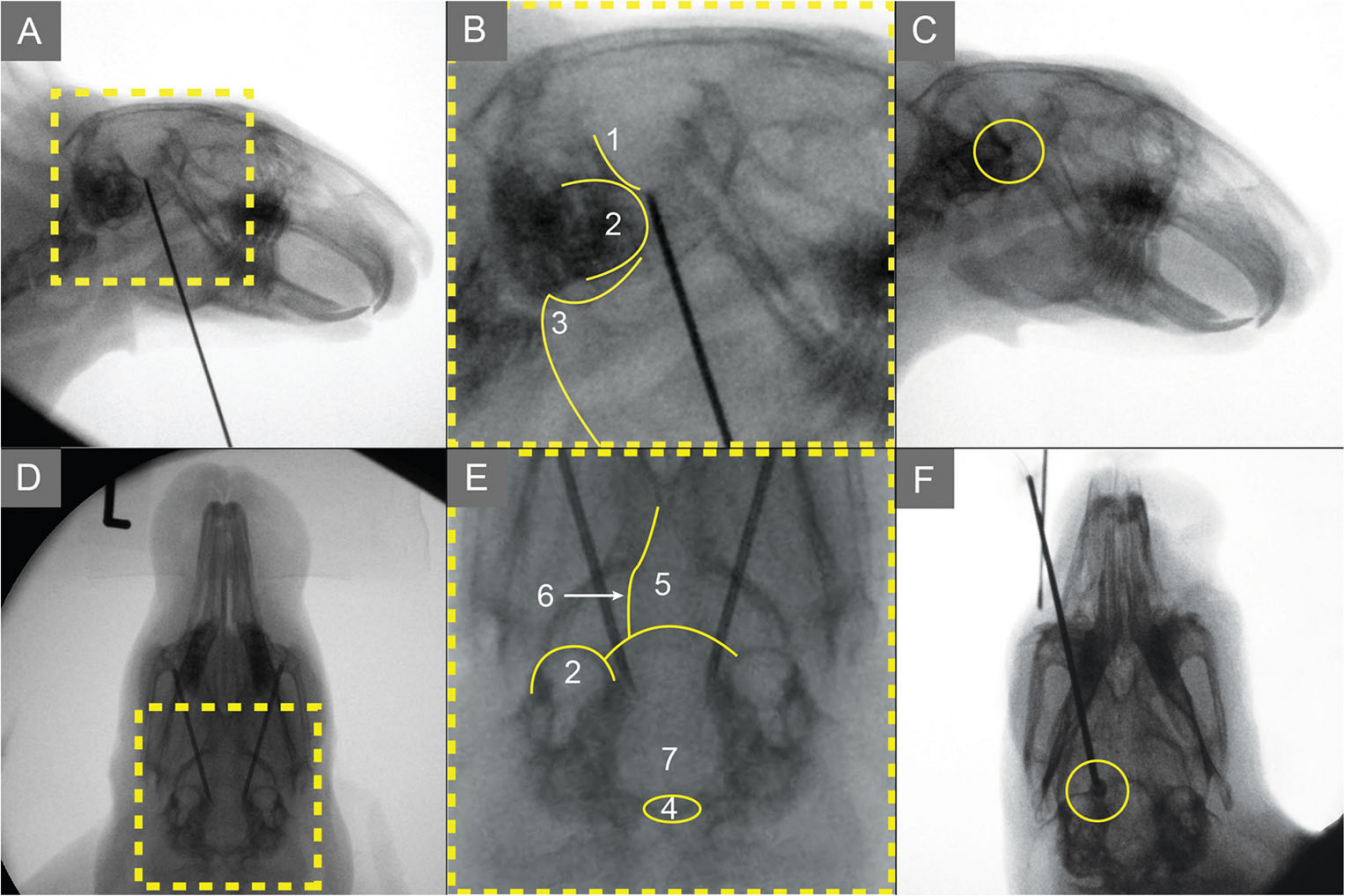
Fluoroscopy is used to confirm correct needle placement while puncturing a New Zealand white rabbit. A and B: Sagittal projection of the head with the needle advanced maximally into the trigeminal cistern (TCI). Further advancement would cause a perforation of the dura mater. D and E: The same head in a dorsoventral projection. Correct needle placement can be confirmed only by checking both planes. C: Simulation of glycerol rhizolysis by injecting contrast agent into the TCI. The radiological control shows the remaining contrast agent 10 min after the injection. F: Correct placement of a balloon catheter. 1, osseous cerebellar tentorium; 2, tympanic cavity; 3, mandibular angular process; 4, foramen magnum; 5, basisphenoid bone; 6, pterygoid bone; 7, occipital bone

The entry of the cannula is precisely at the inner rim of the lower jaw. The cannula is advanced at a 45 degree angle to the PI-Line in the direction of the FO.. Under sagittal fluoroscopy the needle is advanced with an angle of 10 to 15° to the dorsoventral plane (×1). Correct needle placement should be assessed in the dorsoventral (×1) as well as sagittal (×3) plane as shown in Fig. 4A, B, D and E.

#### Source of error

It is important to advance the needle without any resistance. When it is felt, fluoroscopy should confirm correct needle placement. If the cannula is placed too far lateral from the FO, it can be entangled in the poriferous base of the sphenoid bone. Respectively, it can be blocked in a small prominence of the middle cerebral fossa that partly overlaps laterally with the FO (Fig. 3B). In these cases, the position of the cannula can be adjusted more medially. If the cannula passes abruptly through the FO, there is a possibility of perforation of the dura mater and temporal lobe as seen in Fig. 3C.

#### Percutaneous balloon compression (BC)

For the simulation of BC we used Fogarty® No.2 embolectomy catheters of different brands (Edwards Lifesciences™, LeMaitre®). These catheters have short balloon and catheter tip lengths but still exceed the length of the TCI (4.07 ± 0.8 mm).

**Fig. 5:**
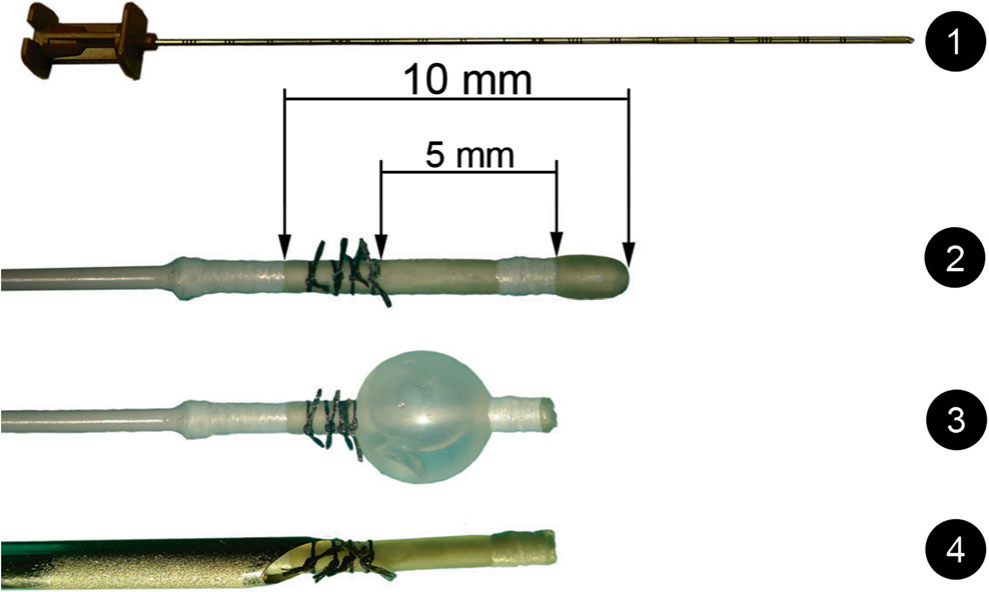
Modified equipment for percutaneous balloon compression (BC). 1: Guiding cannula for BC: Angiotech® CAN 16G 10 cm 2) Fogarty® no. 2 embolectomy catheter with a proximal ligated balloon and (3) distal tip cut for reduction of length. (4) It is important to mark the catheter length to be inserted into the trigeminal cistern in order to accomplish an exact advancement of the balloon out of the cannula tip

Therefore, we modified the LeMaitre® catheter as shown in Fig. 5. Under the operating microscope, the proximal part of the balloon was ligated with four 7–0 Ethicon® ligatures. The catheter tip was cut. Via this method, we could achieve a balloon length of less than 5 mm. As a guiding cannula, a 16-G Angiotech® biopsy needle was used. The Edwards Lifesciences™ catheter has a balloon and tip length of 7 mm and is overall more flexible. No modifications were used. As a guiding cannula, a 14-G Vasofix® Safety cannula was used. Before starting the procedure, it is important to mark the catheter length to be inserted into the TCI to accomplish an exact advancement of the balloon out of the cannula tip. The guiding cannula tip is placed at the entrance of the FO. Afterwards, the balloon catheter is advanced until the balloon completely exits the cannula, which is verified by the previously made mark. The balloon cannot be inflated until its optimal position has been reached. Otherwise the sharp guiding cannula could damage the balloon. Correct placement is again verified by fluoroscopy (Fig. 4F). After the BC has been achieved, the balloon is deflated and removed together with the guiding cannula.

#### Percutaneous glycerol rhizolysis (GR)

NWRs have a closed TCI, and GR can be simulated. We injected up to 0.5 ml of contrast agent (Jopamiro®) and confirmed radiological permanence in the TCI after 5 to 10 min in four NWRs (Fig. 4C). Only small amounts of contrast agent are applicable, and most of the 0.5 ml we injected was lost in the empty space of the cannula or spread out of the TCI into the posterior fossa or back out of the FO.

## Discussion

To better understand the histopathological changes that occur after percutaneous surgical procedures in TN patients, animal experimentation is required. To this day two animal models that reproduce these procedures in mongrel dogs and NWR have been published. Kanpolat described and accurately illustrated the technique used in dogs [10]. But because most European countries prohibit animal experimentation with dogs, in our study we focused on the NWR model introduced by Preul et al. in 1990 [16]. Rabbits seem to be the most appropriate animals for percutaneous procedures. The advantages here are obvious: (1) rabbits are frequently used in experimental research; (2) their cost for experimental research is relatively low; (3) they have an adequately sized FOs; (4) harvesting their fairly large trigeminal nerves is easily carried out; (5) their use for experimental research is not prohibited by law in most European countries; (6) dropout rates in former studies were acceptable [12, 16].

Our initial idea was to perform a histopathological examination after BC. However, when we carried out our preliminary tests on NWR cadavers, we found that the insertion of a needle to the FO as well as further BC had some difficulties. First, our prior knowledge of the anatomy of the trigeminal system in rabbits was very limited, and to our knowledge exact measurements and anatomical descriptions of the middle cranial fossa in rabbits have not been published. Second, in preexisting studies the technique of the puncture itself, the positioning of the animal, landmarks to guide the insertion of the needle under fluoroscopy as well as used materials were inadequately described and illustrated to allow reproduction. To answer these questions in the present study, we obtained accurate morphometric measures in fresh NWR cadavers as well as on rabbit CT scans. Histological proof of the presence and location of the TG was given. The technique of the three most common percutaneous procedures at the TG of rabbits has been described in detail.

### Percutaneous balloon compression (BC)

We found that compression of the TG in the TCI without damaging nearby structures is possible but hampered by inadequately sized equipment. It should be noted that Fogarty® no. 2 embolectomy catheters differ in balloon and catheter tip size, shape and rigidity per different supplier. Even by modification of the Fogarty® no. 2 catheter to a minimal balloon length of 5 mm, its large size was not optimal for BC in rabbits. All three previously reported BC experiments [3, 12, 16] used Fogarty® no. 2 embolectomy catheters with tip lengths larger than the TCI. An adaptation of the catheter to the anatomical conditions was not described by the authors. Furthermore, they used catheter insertion depths beyond the FO of 4–6 mm and 3–5 mm, respectively, while in our measurements the possible insertion depths should not exceed 4.07 ± 0.8 mm. Here, it has to be stressed that Preul et al. as well as Brown et al. used larger NWRs (2.5–3.9 kg/2.5–4.4 kg vs. 1.3–2.9 kg), which could imply a larger TCI. Brown et al. [3] observed that the catheter tip was advanced into the TNC and assumed that trigeminal nerve root damage should be considered as one of the mechanisms involved in the physiopathological effects of BC. We found that the TNC (which is referred to as porus trigeminus in Preul’s work [16]) is a tight channel whose axis is at an acute angle with the axis of the FO. The relatively rigid catheter we used did not change the direction after introduction into the TCI as shown by postoperative dissections.

The extent to which BC in rabbits is comparable to percutaneous procedures in human beings still needs to be assessed. This is not only due to the absence of disease in the animals, but also due to the different anatomy in rabbits. Their TG is nearly completely embedded in bone, as shown in Fig. 2. Nevertheless comparable compression pressures between 829 and 1179 mmHg in NWR [12, 16] and 1140–1216 mmHg in humans [2] seem feasible.

### Percutaneous glycerol rhizolysis (GR)

Among percutaneous procedures, the effect of GR on the nervous system is probably the most commonly studied and at the same time controversially discussed [1, 4, 6–8, 13, 15, 17, 18, 20]. Furthermore, it remains necessary to clarify which fibers are damaged by different concentrations of glycerol.

In our study, we managed to inject contrast agent into the TCI. The contrast agent remained in place for longer than 10 min; 0.5 ml was used, which may seem a lot since Hakanson described 0.2–0.4 ml in humans [6]. Far smaller amounts are possible in rabbits, but we encountered loss of contrast agent into the posterior fossa as well as residues in the cannula in all four cases.

So far, the study of Isik et al. [8] is the only glycerol experiment that used a percutaneous technique at the TG of dogs. The technique used is analogous to the surgical technique introduced by Hakanson [6] in humans. Even here, the experiment cannot be adopted one to one to humans because, according to Isik et al. [8], the TCI is missing in dogs. Thus, glycerol was injected topically into the TG and not into a cistern.

### Percutaneous radiofrequency thermocoagulation (TC)

TC, developed by Sweet [19], can be easily reproduced in rabbits. He proposed that the main mechanism of TC in TN is the selective destruction of thin and non-myelinated A-δ and C fibers at 60–80 °C, which would interrupt pain transmission while maintaining sensitivity. This assumption is contradicted by a study by Kanpolat [11] who carried out TC in dogs and observed no restriction to certain fibers. Of interest and important for future experiments was the finding that in both percutaneous experiments with dogs accidental puncture of the internal carotid artery occurred [8, 11]. This is another reason for preferring rabbits over dogs for an experimental model. Puncture of the internal carotid artery in rabbits is nearly impossible because of a more caudally located artery and the interposed hard basisphenoid bone. Nevertheless, we do not advise the use of smaller rabbits because of a higher risk of accidental puncture of the maxillary artery, which enters the foramen alare caudale (FAC) in the vicinity of the FO.

### Limitations

Morphometric measurements in unfixed cadavers always feature a bias due to quickly occurring cell death. Therefore, we only used fresh NWR cadavers and performed dissections in a fast and standardized way.

## Conclusion

Percutaneous procedures at the TG of rabbits under fluoroscopy were described in detail after carrying out morphometric measurements. This technique enables us to perform percutaneous operations at the TG in a reproducible way compared to how they are carried out in humans suffering from TN. Thus, the histological and pathophysiological changes in the trigeminal system after BC, TC and GR can be examined. While TC and GR are easy to reproduce in rabbits, BC, due to the tight conditions in the TCI, remains a challenge that can be overcome with an adequately small balloon catheter size.

Our model provides the possibility to assess the effect of variable parameters, such as coagulation time and temperature in TC, compression time and pressure in BC and alcohol concentration in GR. These parameters have frequently been discussed in the literature, but mainly by comparing clinically effects and not by comparing morphological changes on the nerve. This standardized model may allow a better understanding of the mechanisms involved and verify whether and which fibers are affected by ablative procedures. We expect that in the future and based on our detailed descriptions of a rabbit model, the knowledge gained will help improve the surgical treatment results and prognosis of percutaneous procedures on the TG.

## Electronic supplementary material

Supplement Three-dimensional skull reconstruction of a rabbit in a lateral view. A “starting position” to enable reproducibility of CT measurements was defined. Intersection point [where all sagittal (×3), dorsoventral (×1) and transverse (×2) planes are perpendicular to each other] is the foramen ovale (FO). In ×3 the head has to be rotated to such a degree that ×1 lies parallel to the PI line, defined as a connecting line between the rostral part of the incisive bone and the external occipital protuberance (JPEG 132 kb)

Supplement 2 Histological cuts through the trigeminal nerve of a New Zealand white rabbit confirms the localization of the trigeminal ganglion. (A) shows a sketch of the following structures: brainstem (1), trigeminal nerve root (2), trigeminal ganglion (3), peripheral nerves with a longitudinal and transverse cut: mandibular nerve V3 (4), maxillary nerve V2 and ophthalmic nerve V1 (5). (B) Bright trigeminal ganglion (SSB staining, 100× magnification) at the left side with darker, more myelinated fibers of V1 and V2 at the upper right. (C) Peripheral nerve, in this case V3 in a longitudinal section (H&E staining, 200× magnification). (D) The trigeminal nerve root enters the trigeminal ganglion coming from the lower right side (SMI staining, 200× magnification). The brown ganglion cells can clearly be depicted from the bluish surrounding glial cells (JPEG 2966 kb)

Supplement 3 Anatomical dissection of a New Zealand white rabbit with particular consideration of the middle cranial fossa and the trigeminal ganglion (TG). A legend of anatomical structures with matching numbers is given in Fig. 5. Illustrations are shown in a dorsal view. **A** Skin incision; **B** and **C** trepanation. **D** After removal of the calvaria and dura mater the brain is visible. **E** and **F** Starting rostrally a cerebrectomy is performed. The following structures are cut: optic chiasm (1), oculomotor nerve (8), trochlear nerve, abducens nerve and trigeminal nerve root (4). **G** After removal of the brain the middle cranial fossa can be inspected. **H** The dura mater encephali at the base (15) of the middle cranial fossa is removed. **I** Ophthalmic nerve and maxillary nerve (6 + 7) stretch from the orbital fissure (OF, 19) to the TG. The TG is covered in the picture by a puncturing cannula (*). The cannula was inserted through the foramen ovale (FO), following the same direction as the mandibular nerve (V3). The roof of the TNC has been previously removed (dotted line). The distal portion of the trigeminal nerve root passes through the TNC (16). **J** A partial removal of the osseous base of the middle cranial fossa was performed to expose V3. The FO (21) is now visible. After rotating V3 rostrally, the motor branch (11) and the sensory branch (5) are depicted. **K** Chasing V3 extra cranially, its peripheral branches are depicted **L** After removal of the trigeminal nerve system, the bone structures can be studied and measured [e.g., distance between FO (21) and TNC (16), which could indicate the possible length of a compressing balloon] (JPEG 4026 kb)

## Abbreviations

BC: Percutaneous balloon compression
EOP: External occipital protuberance (protuberantia occipitalis externa)
FAC: Foramen alare caudale
FO: Foramen ovale
GR: Percutaneous glycerol rhizolysis
NWR: New Zealand white rabbits
OF: Orbital fissure (fissura orbitalis)
PI-Line: Line between the external occipital protuberance and incisive bone
TC: Percutaneous radiofrequency-thermocoagulation
TCI: Trigeminal cistern (cisterna trigemini)
TG: Trigeminal ganglion (ganglion trigeminale)
TN: Trigeminal neuralgia
TNC: Trigeminal nerve canal (canalis nervi trigemini)
V1: Ophthalmic nerve (nervus ophthalmicus)
V2: Maxillary nerve (nervus maxillaris)
V3: Mandibular nerve (nervus mandibularis)
×1: Dorsoventral plane
×2: Transverse plane
×3: Sagittal plane
