# Supplementary figures and images for: An experimental animal model for percutaneous procedures used in trigeminal neuralgia
Source: Acta Neurochir (Wien). 2017 Apr 10;159(7):1341–8. doi: 10.1007/s00701-017-3162-8 (PMC5486611; doi:10.1007/s00701-017-3162-8)

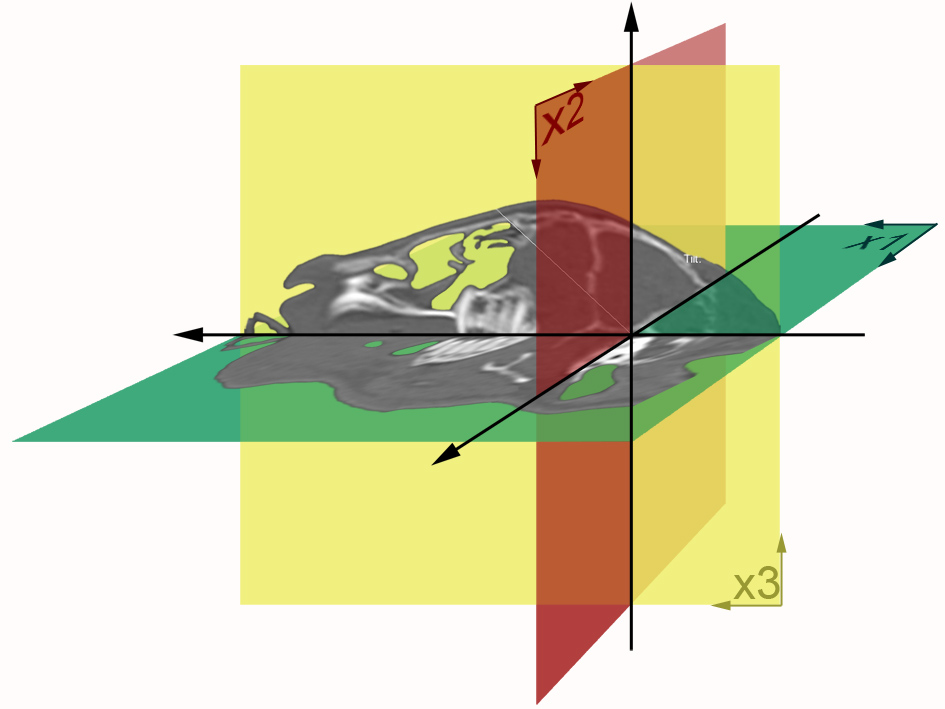

Supplement: Supplementary file 1 — Three-dimensional skull reconstruction of a rabbit in a lateral view. A “starting position” to enable reproducibility of CT measurements was defined. Intersection point [where all sagittal (×3), dorsoventral (×1) and transverse (×2) planes are perpendicular to each other] is the foramen ovale (FO). In ×3 the head has to be rotated to such a degree that ×1 lies parallel to the PI line, defined as a connecting line between the rostral part of the incisive bone and the external occipital protuberance (JPEG 132 kb) [file 701_2017_3162_MOESM1_ESM.jpg]

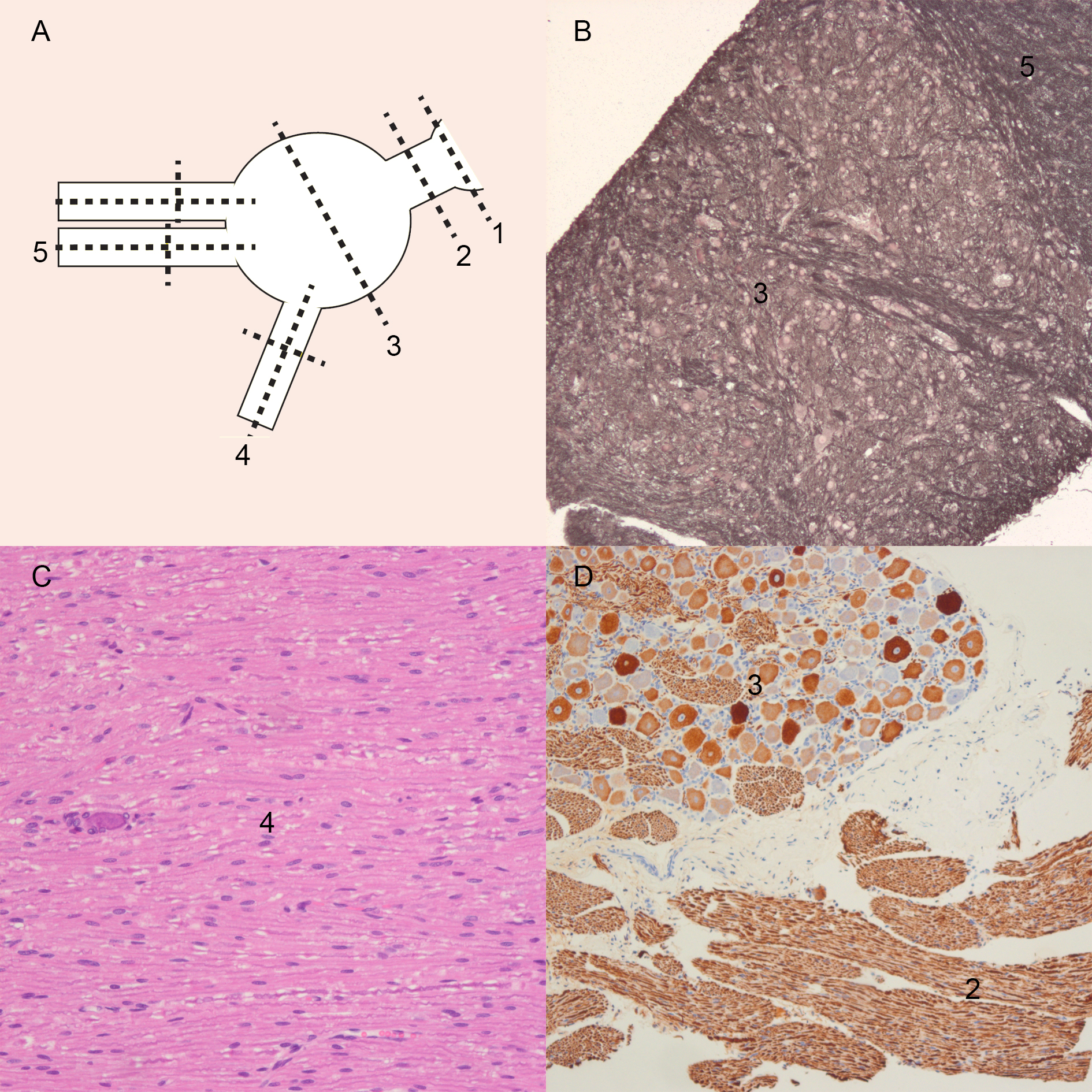

Supplement: Supplementary file 2 — Histological cuts through the trigeminal nerve of a New Zealand white rabbit confirms the localization of the trigeminal ganglion. (A) shows a sketch of the following structures: brainstem (1), trigeminal nerve root (2), trigeminal ganglion (3), peripheral nerves with a longitudinal and transverse cut: mandibular nerve V3 (4), maxillary nerve V2 and ophthalmic nerve V1 (5). (B) Bright trigeminal ganglion (SSB staining, 100× magnification) at the left side with darker, more myelinated fibers of V1 and V2 at the upper right. (C) Peripheral nerve, in this case V3 in a longitudinal section (H&E staining, 200× magnification). (D) The trigeminal nerve root enters the trigeminal ganglion coming from the lower right side (SMI staining, 200× magnification). The brown ganglion cells can clearly be depicted from the bluish surrounding glial cells (JPEG 2966 kb) [file 701_2017_3162_MOESM2_ESM.jpg]

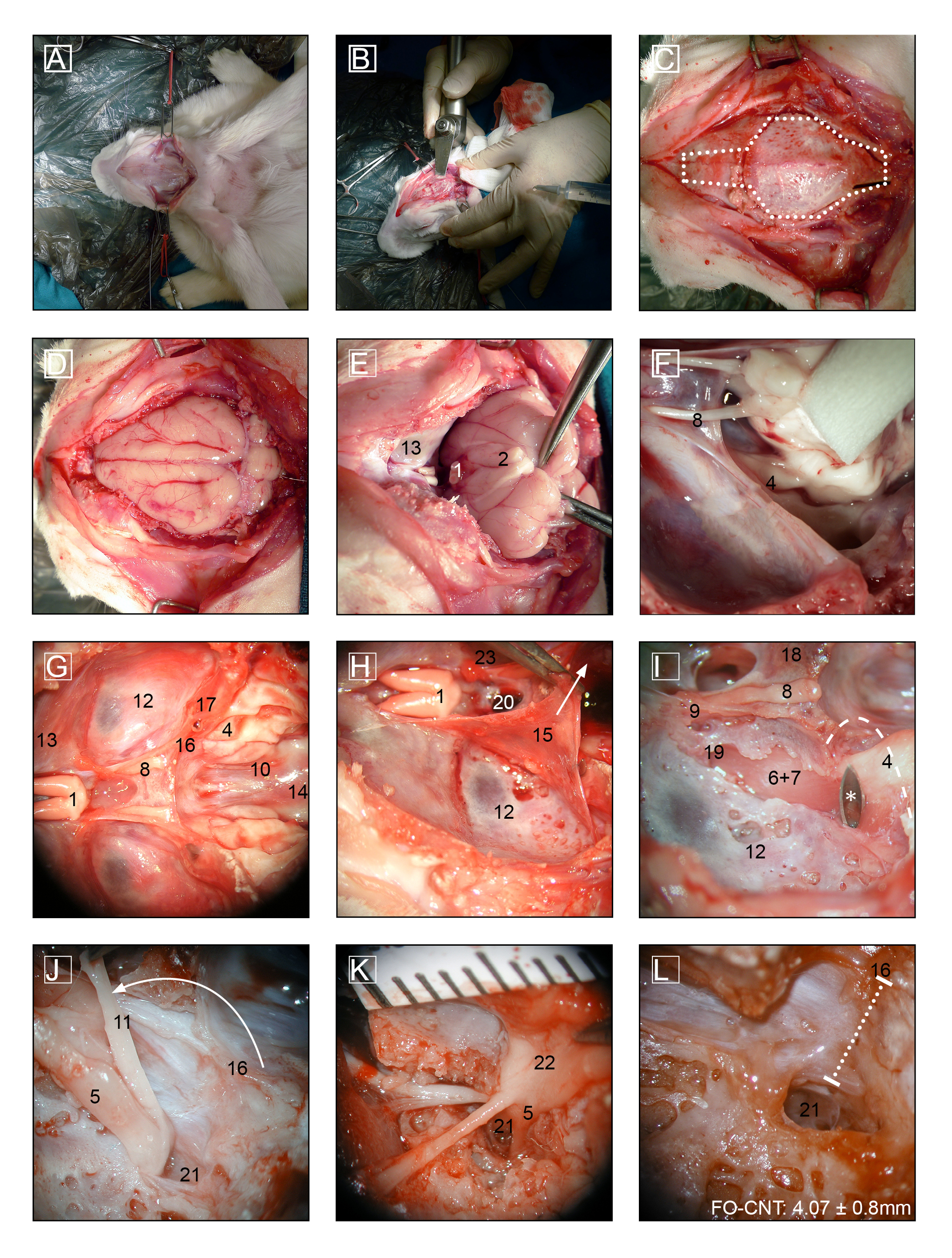

Supplement: Supplementary file 3 — Anatomical dissection of a New Zealand white rabbit with particular consideration of the middle cranial fossa and the trigeminal ganglion (TG). A legend of anatomical structures with matching numbers is given in Fig. 5. Illustrations are shown in a dorsal view. A Skin incision; B and C trepanation. D After removal of the calvaria and dura mater the brain is visible. E and F Starting rostrally a cerebrectomy is performed. The following structures are cut: optic chiasm (1), oculomotor nerve (8), trochlear nerve, abducens nerve and trigeminal nerve root (4). G After removal of the brain the middle cranial fossa can be inspected. H The dura mater encephali at the base (15) of the middle cranial fossa is removed. I Ophthalmic nerve and maxillary nerve (6 + 7) stretch from the orbital fissure (OF, 19) to the TG. The TG is covered in the picture by a puncturing cannula (*). The cannula was inserted through the foramen ovale (FO), following the same direction as the mandibular nerve (V3). The roof of the TNC has been previously removed (dotted line). The distal portion of the trigeminal nerve root passes through the TNC (16). J A partial removal of the osseous base of the middle cranial fossa was performed to expose V3. The FO (21) is now visible. After rotating V3 rostrally, the motor branch (11) and the sensory branch (5) are depicted. K Chasing V3 extra cranially, its peripheral branches are depicted L After removal of the trigeminal nerve system, the bone structures can be studied and measured [e.g., distance between FO (21) and TNC (16), which could indicate the possible length of a compressing balloon] (JPEG 4026 kb) [file 701_2017_3162_MOESM3_ESM.jpg]
